# Effects of propofol versus sevoflurane on surgical field visibility during arthroscopic rotator cuff repair: a randomized trial

**DOI:** 10.1186/s12871-024-02403-1

**Published:** 2024-01-17

**Authors:** Wenchao Yin, Chenzhu Yin, Wencan Wang, Rao Peng, Li Su, Peiyu Li

**Affiliations:** Department of Anesthesiology, Sichuan Provincial Orthopedic Hospital, No. 132 West First Section First Ring Road, Chengdu, 610041 Sichuan China

**Keywords:** Propofol, Sevoflurane, Rotator cuff injuries, Arthroscopy, Surgical field visibility, Blood loss, surgical

## Abstract

**Background:**

During arthroscopic rotator cuff repair (ARCR), clear surgical field visibility (SFV) is the basis of successful surgery, but the choice of anesthesia maintenance drugs may have different effects on SFV. In this study, we aimed to compare the effects of propofol- and sevoflurane-based general anesthesia on SFV in patients undergoing ARCR.

**Methods:**

Patients (*n* = 130) undergoing elective ARCR in the lateral decubitus position were randomized into either the propofol group or sevoflurane group (65 per group). The duration of surgery and increased pressure irrigation (IPI), Boezaart score, rocuronium consumption and usage of remifentanil were recorded. The time of both spontaneous respiration recovery and extubation and the incidences of postoperative nausea and vomiting and agitation were also recorded.

**Results:**

The Boezaart score, duration of IPI and ratio of the duration of IPI to the duration of surgery (IPI/S ratio) were similar between the groups (*P* > 0.05). Rocuronium consumption, number of patients requiring remifentanil infusion and total remifentanil consumption were significantly lower in the sevoflurane group (*P* < 0.05). The spontaneous respiration recovery time was significantly longer in the propofol group (*P* < 0.05), but there were no differences in the extubation time between the groups(*P* > 0.05).

**Conclusions:**

Compared with propofol, sevoflurane provides equally clear SFV while improving the convenience of anesthesia maintenance in ARCR patients with interscalene plexus (ISB) combined with general anesthesia.

**Trial registration:**

This single-center, prospective, RCT was retrospective registered at Chinese Clinical Trial Registry with the registration number ChiCTR2300072110 (02/06/2023).

## Introduction

During arthroscopic rotator cuff repair (ARCR), intra-articular haemorrhage is the principal factor influencing surgical field visibility (SFV) and should be controlled carefully; otherwise, diagnosis and repair may not be accomplished [1]. Optimal SFV is essential to achieve the most accurate, stable and functional ARCR [2]. Three techniques have been shown to be effective in controlling bleeding during ARCR, including lowering blood pressure(BP), increasing irrigation pressure, and surgical haemostasis [3]. However, lowering BP may increase the risk of organ hypoperfusion, while increasing irrigation pressure may aggravate fluid extravasation and result in tissue oedema [3–5]. Therefore, the use of these techniques for bleeding control must be reasonable, and sometimes other additional interventions must inevitably be considered. In previous studies, researchers have tried to explore other methods to improve SFV, and have obtained some satisfactory findings [3, 6]. At present, continuous administration of irrigation fluid containing diluted epinephrine or norepinephrine is a well-known and commonly used method that has been demonstrated to be effective, but it is associated with an increased incidence of hypotensive and bradycardic events [6].

Propofol and sevoflurane are two commonly used general anesthetics that have an established safety track record lasting over 30 years [7]. However, the choice between propofol- and sevoflurane-based general anaesthesia may sometimes affect intraoperative bleeding and result in a different surgeon experience [8–11]. It has been validated that propofol- is superior to sevoflurane-based anesthesia during nasal surgeries with general anesthesia in terms of bleeding control, SFV and surgeon satisfaction [8, 9, 11]. Similarly, when patients undergo arthroscopic shoulder surgery with interscalene plexus block (ISB), the selection of propofol target‑controlled infusion (TCI) over sevoflurane inhalation for sedation could decrease BP and bleeding, while improving visualization [10]. However, as patients receive ISB combined with general anesthesia with controlled hypotension for arthroscopic shoulder surgery, it is not currently known whether propofol-based anesthesia is still superior to sevoflurane-based anesthesia in terms of SFV. We hypothesized that propofol-based anesthesia may provide better SFV than sevoflurane-based anesthesia during ARCR with controlled hypotension. Therefore, we conducted a randomized study to assess the effects of sevoflurane on SFV (modified Boezaart score), the duration of surgery and increased pressure irrigation (IPI) during ARCR. In addition, the consumption of analgesics and muscle relaxants and patient recovery were also included in the analysis.

## Methods

### Ethics

This study was prospective and randomized controlled, which conducted in accordance with the Declaration of Helsinki tenets. It approved by the Ethics Committee of Sichuan Provincial Orthopedic Hospital on August 12, 2020 (reference KY2020-001-01), and retrospective registered at www.chictr.org.cn with the registration number ChiCTR2300072110.

### Study population

The study adhered to the Consolidated Standards of Reporting Trials (CONSORT) guidelines [12]. Patients agaed 40 to 60 years who underwent elective ARCR with American Society of Anesthesiologists (ASA) Physical Status I or II were considered eligible participants. We excluded patients who declined to participate in the study and those with coagulation disorders or continuous anticoagulant administration, preexisting cardiovascular diseases such as hypertension and coronary heart disease, diabetes mellitus, respiratory disorders, hyperlipidemia, a body mass index (BMI) > 30 kg/m^2^ or ＜19 kg/m^2^ and contraindications to ISB. Participating patients signed written informed consent forms the day before the surgery. A computer-generated, block randomization schedule at a 1:1 ratio was used, and the sequentially numbered, opaque, sealed envelopes containing the assignments were prepared by a investigator who was not involved in any other parts of the study. Enrolled patients were allocated to either the propofol or sevoflurane group according to the assignments, which were concealed in the envelope and opened by the anesthesiologist immediately after patients arrived in the operating room .

### Procedures

In this study, both the ISB and general anesthesia were performed by the same experienced anesthesiologist, and all surgeries were performed in the lateral decubitus position by a senior surgeon. The operating room temperature was maintained at 21℃ ± 1℃, and all IV and irrigation fluids were administered at this temperature. All patients received forced-air warming, and the heater working temperature was set at 43℃ [13]. A gravity irrigation system that consisted of 2 (3-L) saline bags suspended 70 cm above the surgical shoulder joint was used in this study to create an inflow pressure for adequate intra-articular visualization. There was no additional agent in the irrigation fluid. To improve visualization, an increase in the irrigation pressure by raising the height of the saline bags for brief periods was permitted when bleeding was difficult to control.

On arrival at the holding area, patients underwent standard ASA monitoring and received IV Ringer’s solution at a rate of 5 ml.kg^− 1^.h^− 1^. Then the anesthesiologist performed the ISB and contralateral radial artery catheterization under ultrasound guidance. After the brachial plexus roots were visualized using a high-frequency linear ultrasound transducer (Navi U, Wisonic Medical, China), a 22G 0.71 × 50 mm needle (Stimuplex D, B. Braun, Germany) was inserted and 20 mL of 0.2% ropivacaine was injected around the nerve roots under ultrasound observation [14]. The arterial pressure was measured continuously by a radial artery catheter throughout the operation and the pressure transducer remained fixed at the level of the heart. After confirming the success of the ISB (C5 and C6 dermatomes sensory loss), general anesthesia was induced with propofol (2.0 mg · kg^− 1^), rocuronium (0.8 mg · kg^− 1^), and sufentanil (5.0 µg · kg^− 1^). Patients were intubated with an endotracheal tube and underwent mechanical ventilation until spontaneous respiration was restored.

After intubation, patients in the propofol group received a continuous IV infusion of propofol for anesthesia maintenance (propofol-based anesthesia), and those in the sevoflurane group received inhalation of sevoflurane (sevoflurane-based anesthesia); the bispectral index (BIS) target value in both groups was set from 40 to 60 during the operation. During surgery, the neuromuscular blockade was measured at the adductor pollicis muscle every 5 min using a neuromuscular transmission monitor (BeneVision N17 Mindray monitor, Shenzhen, China), and train-of-four (TOF) ratio of 0 was maintained by intermittent rocuronium injections until the rotator cuff repair was completed. During the operation, the systolic blood pressure (SBP) was maintained between 90 and 100 mmHg by the additional administration of remifentanil or vasopressor (methoxamine or ephedrine). thirty minutes after incision, the heart rate (HR) was recorded as the intraoperative HR. At the beginning of skin closure, the administration of anesthetics was stopped, 5 mg tropisetron was administered by IV, and the fresh gas flow was increased to 6 l/min.

The following information was documented for each patient at the end of the operation: the durations of surgery and IPI, irrigation volume, SFV grading in terms of bleeding (Modified Boezaart score), rocuronium consumption and the usage of remifentanil infusion. The surgeon graded the SFV from 0 to 5 based on the modified Modified Boezaart score scale (Table 1), with 0 denoting the best and 5 denoting the worst visibility [9].

**Table 1:** Modified boezaart score scale

Boezaart score	Description
0	No bleeding
1	Slight bleeding requiring no electrocoagulation
2	Slight bleeding requiring occasional electrocoagulation without threatening the SFV
3	Slight bleeding requiring frequent electrocoagulation and occasional IPI to minimize the threat of bleeding to the SFV
4	Moderate bleeding requiring frequent electrocoagulation and frequent IPI to minimize the threat of bleeding to the SFV
5	Severe bleeding, with frequent electrocoagulation and frequent IPI required, and with the SFV severely threatened, and surgery not possible

SFV: surgical field visibility, IPI: increased pressure irrigation

After the resumption of spontaneous respiration, patients received neostigmine 0.04 mg. kg^− 1^ and atropine 0.015 mg. kg^− 1^ for neuromuscular block reversal. The endotracheal tube was removed when patients opened their eyes in response to verbal instructions and the TOF ratio was＞0.9. Then, patients were transferred to the postanesthesia care unit (PACU) and followed up for 1 h. The time of spontaneous respiration recovery and extubation and the incidence of postoperative nausea, vomiting, and agitation were recorded as patients left the operating room.

### Statistical analysis

The PASS 15 (NCSS, LLC. Kaysville, Utah, USA) was used to calculate the sample size. From the published literature, we determined that the mean SFV scores (Modified Boezaart score) of propofol and sevoflurane were 3.24 and 3.94, and the standard deviations (SDs) were 1.31 and 1.39, respectively [9]. Accounting for a potential 5% dropout rate, 130 patients (65 per group)was considered an acceptable sample size to provide 80% power at a two-sided significance level (α) of 0.05.

All statistical analyses were performed by SPSS statistics 24.0 (IBM Corp, Armonk, NY, USA), and *P* < 0.05 was considered statistically significant. Normally distributed continuous data are reported as the mean ± SD, and Student’s t test was used to compare variables between the groups. Nonnormal distributed data are reported as medians (interquartile ranges), and were compared by the Mann–Whitney U test. We applied χ2 or Fisher exact tests to investigate associations among discrete variables.

## Results

**Fig. 1:**
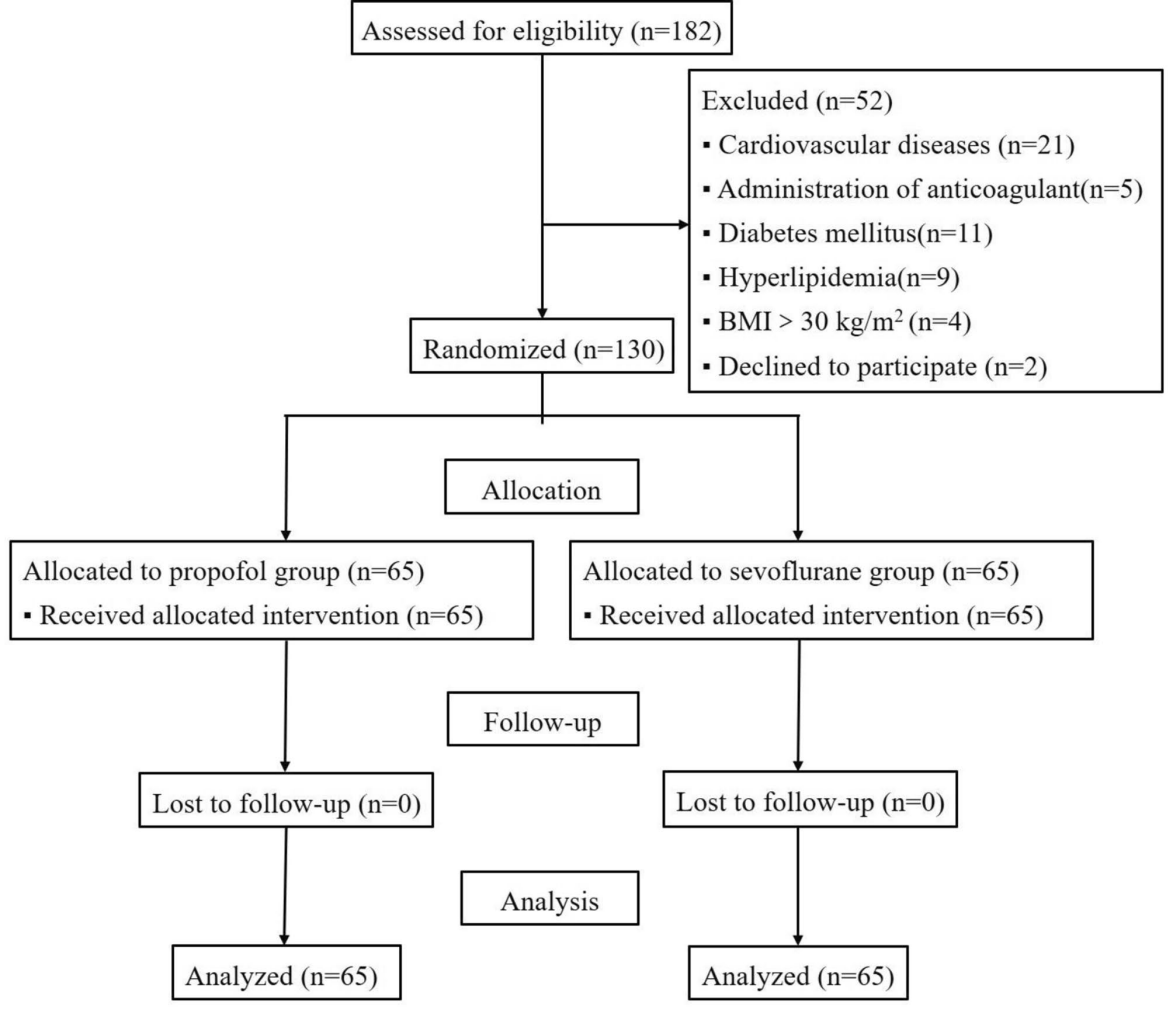
Consolidated standards of reporting trials (CONSORT) flow diagram showing patient progress through the study. BMI: body mass index

From January 2, 2022, to August 10, 2022, 182 patients who were scheduled for ARCR were considered eligible, and 52 patients were excluded before randomization (Fig. 1). In total, 130 patients were enrolled, and randomly allocated to one of the study groups (65 per group). All patients completed the study. The baseline patient and surgical characteristics are summarized in Table 2, and no significant differences were noted between the propofol and sevoflurane groups.

**Table 2:** Patient and surgical characteristics

	Propofol group (*n* = 65)	Sevoflurane group (*n* = 65)	***P*** value
Sex, male/female	31/34	32/33	0.861
Age, y	52.3 ± 5.5	51.5 ± 4.9	0.405
BMI, kg/m^2^	23.9 ± 2.7	23.8 ± 2.8	0.847
NIBP, mmHg			
SBP	125.9 ± 8.9	125.7 ± 10.3	0.891
DBP	72.2 ± 10.1	72.6 ± 10.6	0.859
MBP	90.1 ± 9.5	90.3 ± 10.4	0.930
Preoperative HR, bpm	77.2 ± 10.4	76.8 ± 9.8	0.795
Number of tendons repaired			0.847
1	35	37	
2	23	20	
3	7	8	
Irrigation volume, L	16.8 ± 6.0	16.9 ± 5.6	0.986
Intraoperative blood loss, mL	45.5 ± 25.4	46.3 ± 24.5	0.861

Data are expressed as Mean ± SD. NIBP: non-invasive blood pressure, SBP: systolic blood pressure, DBP: diastolic blood pressure, MBP: mean blood pressure, HR: heart rate, bpm: beats per minute

The modified Boezaart score was applied to assess SFV in terms of bleeding, and it was similar between the groups. The preoperative HR, duration of surgery, duration of IPI and ratio of the duration IPI to the duration of surgery ( IPI/S ratio) were also similar between the groups (Table 3). Compared with the propofol group, in the sevoflurane group, the consumption of rocuronium (98.5 ± 23.5 mg vs. 89.0 ± 18.9 mg; *P* = 0.013), the number of patients requiring remifentanil infusion (50 [76.9%] vs. 16 [24.6%]; *P* < 0.001) and the total remifentanil consumption in patients requiring infusion (317.4 ± 165.5 µg vs. 239.4 ± 91.4 µg; *P* = 0.021) were significantly lower (Table 3).

**Table 3:** Comparison of boezaart score, intraoperative HR, duration of surgery, duration of IPI, rocuronium consumption and remifentanil infusion

	Propofol group (*n* = 65)	Sevoflurane group (*n* = 65)		***P*** value
Modified Boezaart score	1 (1.5)		2 (1.5)	0.456
Intraoperative HR, bpm	67.3 ± 11.2		68.2 ± 10.7	0.632
Duration of surgery, min	82.5 ± 27.3		81.1 ± 24.8	0.758
Duration of IPI, min	5 (7)		6 (6.5)	0.459
IPI/S ratio, %	7.7 ± 5.3		8.7 ± 5.1	0.301
Rocuronium consumption, mg	98.5 ± 23.5		89.0 ± 18.9	0.013
Remifentanil infusion				
Number of patients requiring, n (%)	50 (76.9)		16 (24.6)	＜0.001
Total remifentanil consumption in patients requiring infusion, µg	317.4 ± 165.5		239.4 ± 91.4	0.021
Mean speed of remifentanil infusion, µg/kg/min	0.15 ± 0.05		0.12 ± 0.06	0.118

Data are expressed as Mean ± SD, median (interquartile range), or number (percentages) as appropriate. IPI: increasing pressure irrigation, IPI/S ratio: duration of IP- to- duration of surgery ratio

The spontaneous respiration recovery time was significantly longer in the propofol group than in the sevoflurane group (9.1 ± 5.3 min vs. 6.8 ± 3.4 min; *P* = 0.004), but there was no difference in the extubation time between the groups (12.6 ± 5.9 min vs. 11.8 ± 4.7 min; *P* = 0.389).There were no significant differences in the incidence of nausea, vomiting, or agitation (Table 4).

**Table 4:** Comparison of spontaneous respiration recovery time, extubation time, incidence of nausea, vomiting and agitation

	Propofol group (*n* = 65)	Sevoflurane group (*n* = 65)	***P*** value
Spontaneous respiration recovery time, min	9.1 ± 5.3	6.8 ± 3.4	0.004
Extubation time, min	12.6 ± 5.9	11.8 ± 4.7	0.389
Nausea, n (%)	13 (20.0)	18 (27.7)	0.411
Vomiting, n (%)	9 (13.8)	11 (17.0)	0.809
Agitation, n (%)	5 (7.7)	9 (13.8)	0.397

Data are expressed as Mean ± SD or number (percentages) as appropriate

## Discussion

In this study, we investigated the effects of propofol versus sevoflurane on SFV when used for general anesthesia maintenance during ARCR. Our results showed that the modified Boezaart score and IPI/S ratio were similar between the propofol- and sevoflurane-based anesthesia groups, which indicated that the two anesthesia techniques provide equivalent SFV. In addition, sevoflurane-based anesthesia reduced the demand for intraoperative remifentanil infusion and decreased rocuronium consumption, which makes the administration of anesthesia more convenient.

Rotator cuff tears, a common cause of shoulder pain and even disability, usually occur after the age of 40 years, and more than two-thirds of patients undergoing rotator cuff repair are of working age [15]. ARCR is a useful technique for rotator cuff tears diagnosis and repair, and the quality of SFV is a crucial factor for successful ARCR [1, 2]. Additionally, considering the risk of controlled hypotension in elderly patients, we only enrolled patients aged 40 to 60 years to compare the effects of propofol versus sevoflurane on SFV during ARCR.

Controlled hypotension is the most effective method for controlling bleeding and achieving clear SFV [16]. Although ARCR can be completed under brachial plexus block, general anesthesia or combined anesthesia (brachial plexus block and general anesthesia) [17–21], combined anesthesia is more conducive to the implementation of controlled hypotension [19]. In this trial, we applied combined anesthesia in all patients because it not only improves the patient experience but also facilitates airway management and the implementation of controlled hypotension by anesthesiologists. With the consideration of cerebral perfusion, an SBP of 100 mmHg has been demonstrated to be ideal for optimal visualization in patients undergoing ARCR in the beach-chair position [22]. The lateral decubitus position adopted in the study decreased the risk of cerebral ischemia during ARCR compared with the beach-chair position [23]. As a result, we kept the SBP between 90 and 100 mmHg to attempt to achieve optimal SFV. Nevertheless, during ARCR, the impact of bleeding on SFV must still be reduced by increasing irrigation pressure to provide clear visibility for surgical hemostasis. Of course, the duration of IPI is also restricted because IPI may aggravate irrigation fluid extravasation and increase the risk of airway compromise and respiratory distress [4, 5, 19]. In this study, we recorded the duration of IPI throughout ARCR and calculated the IPI/S ratio. We believe these data can provide an objective assessment for SFV.

Both propofol and inhaled anaesthetics can induce peripheral vasodilation, but the mechanisms of vasodilation are different. Propofol acts by depressing sympathetic tone rather than directly acting on peripheral vascular smooth muscle [8]. However, inhaled anaesthetics cause peripheral vasodilation due to direct relaxation of the pre-capillary sphincters as well as suppression of sympathetic tone, thereby promoting more blood flow to the surgical field [8, 24]. As one of the most commonly used inhaled anesthetics, sevoflurane has also exhibited considerable potential to dilate microvessels. At the comparable anesthesia depth (BIS value) and/or BP level, sevoflurane-based anesthesia shows stronger microvasodilatory effects than propofol-based anesthesia [25–27]. Multiple studies have demonstrated that propofol-based anesthesia can effectively reduce bleeding and improve SFV during endoscopic sinus surgery and rhinoplasty when compared with sevoflurane-based anesthesia [8, 9, 11]. During middle ear microsurgery, anesthesia maintained with propofol/remifentanil provides better SFV than anesthesia maintained with desflurane/remifentanil [28]. During ARCR under ISB, Tantry et al. [10] also found that propofol TCI was superior to sevoflurane inhalation in terms of the clarity of SFV, but they attributed this finding to the fact that sedation by propofol TCI could decrease the intraoperative BP to a greater extent. In our study, we applied controlled hypotension in all patients to eliminate the confounding factor of BP in the SFV study and found that the visibility score (modified Boezaart score) and IPI/S ratio were similar between the groups. In addition, Nair et al. [29] demonstrated that a slow HR is beneficial for venous capacitance vessel filling and improves SFV by decreasing venous oozing in the surgical field. However, we found no difference in the intraoperative HR between the two groups. These findings revealed that sevoflurane-based anesthesia and propofol-based anesthesia provide equally clear SFV during ARCR.

Rotator cuff tears are often accompanied by muscles atrophy and tendons contract [30, 31], which requires deep muscle relaxation for the repair of ruptured tendons. Additionally, during ARCR, deep muscle relaxation is also required for the maintenance of a sufficient operating space. In our study, sevoflurane-based anesthesia decreased the consumption of rocuronium, possibly because sevoflurane increases the intensity and duration of action of neuromuscular blocking agents [32]. Sevoflurane inhalation can also inhibit the transmission of nociception and reduce the demand for opioids [32]. Due to these potential effects, we found that the spontaneous respiration recovery time was shortened after sevoflurane-based anesthesia. However, the extubation time was similar between the groups, which might be attributed to faster recovery after propofol-based than sevoflurane-based anesthesia [9]. Sevoflurane is associated with postoperative nausea, vomiting and agitation [9, 33]. In our study, although the incidences of postoperative nausea, vomiting, and agitation were higher for the sevoflurane-based anesthesia group than for the propofol-based anesthesia group, no significant differences were noted. The best explanation for these findings appears to be that more patients received remifentanil infusion, and the consumption of remifentanil was higher during propofol-based anesthesia, which potentially led to postoperative nausea and vomiting [34]. In addition, since all patients received ISB, emergence agitation was seldom observed in this study. Taken together, these findings indicated that anesthesia maintained with sevoflurane is more convenient for anesthesiologists than maintained with propofol during ARCR and has no obvious influence on the incidence of unpleasant postoperative patient experiences.

To our knowledge, this is the first report to use the IPI/S ratio to indirectly reflect the quality of SFV. Although this indicator is much more objective and exhibits a good correlation with the Modified Boezaart score, it seems to not only represent SFV. During previous ARCR, we observed surgeons occasionally expanding the operating space by IPI. This may be the main limitation of the present study. We attempted to minimize it by monitoring neuromuscular blockade and maintaining deep muscle relaxation. In addition, based on our study experience, it is difficult for some young and healthy patients to achieve satisfactorily controlled hypotension after anesthesia by administration of sevoflurane or propofol alone. In this situation, remifentanil infusion is essential, but the administration of remifentanil may interfere with the comparison of postoperative nausea and vomiting incidence between propofol- and sevoflurane-based anesthesia.

## Conclusion

During ARCR under ISB combined with general anesthesia and controlled hypotension, propofol-based anesthesia and sevoflurane-based anesthesia provide equally clear SFV, but sevoflurane-based anesthesia is more convenient for anesthesiologists to administer.

## Data Availability

The datasets used and analyzed during the current study are available from the corresponding author upon reasonable request.

## Abbreviations

ARCR: Arthroscopic rotator cuff repair
SFV: Surgical field visibility
IPI: Increased pressure irrigation
ISB: Interscalene plexus block
BP: Blood pressure
TCI: Target‑controlled infusion
SBP: Systolic blood pressure
PACU: Postanesthesia care unit
ASA: American Society of Anesthesiologists
BMI: Body mass index
HR: Heart rate
bpm: Beats per minute
